# Cyclodextrin–silica hybrid materials: synthesis, characterization, and application in pesticide aqueous removal

**DOI:** 10.3389/fchem.2024.1450089

**Published:** 2024-08-29

**Authors:** Estefanía Baigorria, Lucas Bragança Carvalho, Luciana Matos Alves Pinto, Leonardo Fernandes Fraceto

**Affiliations:** ^1^ Institute of Science and Technology, São Paulo State University, Sorocaba, Brazil; ^2^ Materiales Compuestos Termoplásticos (CoMP), Instituto de Investigaciones en Ciencia y Tecnología de Materiales (INTEMA), Consejo Nacional de Investigaciones Científicas y Técnicas, Universidad Nacional de Mar del Plata (UNMdP), Buenos Aires, Argentina; ^3^ Chemistry Department, Natural Sciences Institute, Universidade Federal de Lavras, Lavras, Brazil

**Keywords:** adsorption, nanocomposites, pesticides, environmental remediation, water remediation

## Abstract

**Introduction:**

Overusing and misusing pesticides, including paraquat (PQ), have led to numerous environmental contamination complications. PQ is an emerging bio-accumulative contaminant that is present in environmental aqueous matrices. Adsorption techniques are part of a set of technologies applied in ecological remediation, known for their high effectiveness in removing aqueous PQ. A study of the PQ adsorption capacity of three cyclodextrin–silica nanocomposites (α-CDSi, β-CDSi, and γ-CDSi) from contaminated waters is presented in this paper.

**Methods:**

The cyclodextrin–silica nanocomposites were synthesized via an esterification reaction between the inorganic matrix and cyclodextrins (CDs) (α, β, and γ) and were characterized physicochemically by spectroscopic, thermal, and surface methods. Their PQ removal performance from contaminated aqueous media was studied under different experimental conditions.

**Results and Discussion:**

The results showed a fast adsorptive response in removal treatment studies over time. Adsorption capacities of 87.22, 57.17, and 77.27 mg.g^−1^ were found for α-CDSi, β-CDSi, and γ-CDSi, respectively, at only 30 min of treatment. Thermodynamic studies indicated spontaneous and exothermic adsorption processes. The removal assays responded mainly to physisorption mechanisms with contributions from chemisorption mechanisms. Spectroscopic assays showed a strong interaction of PQ with the adsorbents used. Innovative CDSi nanocomposites have proven to be highly efficient in applying aqueous PQ remediation, thus proving to be sustainable adsorbents of contaminants of emerging importance worldwide.

## 1 Introduction

Research breakthroughs have led to the development of various hybrid materials with significant novel properties (Kausar, 2018; Li et al., 2021). Generating new materials with a combination of diverse properties allows for obtaining a single structure with superior properties to those of its components (Kausar, 2018). Inorganic–organic hybrid materials possess applications in numerous fields based on their unique physical, chemical, and mechanical properties (Kausar, 2018; Li et al., 2021). In recent years, the application of hybrid materials as adsorbents in the aqueous remediation field has been growing (Kausar, 2018; Li et al., 2021; Baigorria and Fraceto, 2022a).

Silica (Si) is an inert inorganic polymer with promising adsorbent properties (Morin-Crini et al., 2019). Silica gel has a high surface area and porosity, its toxicity is zero, and it is one of the lowest-cost inorganic materials (Morin-Crini et al., 2019). Silica has wide possibilities of functionalization on its surface due to the presence of the silanol group (Bragança Carvalho et al., 2019; Morin-Crini et al., 2019). The silanol groups allow the immobilization of various ligands on the silica structure, such as cyclodextrins (CDs), thus obtaining new adsorbent hybrid materials (Bragança Carvalho et al., 2019; Morin-Crini et al., 2019). In recent years, increasing reports have been found on various applications of silica and cyclodextrin hybrid materials as adsorbents in environmental remediation treatments (Bragança Carvalho et al., 2019; Junthip, 2019; Junthip et al., 2019; Morin-Crini et al., 2019; Martwong et al., 2021; Martwong et al., 2022; Sun et al., 2022).

CDs are cyclic oligosaccharides formed by the enzymatic degradation of starch and can form inclusion complexes with numerous molecules (Bragança Carvalho et al., 2019; Morin-Crini et al., 2019; Liu et al., 2020). The α-CDs, β-CDs, and γ-CDs (six, seven, and eight glucose units, respectively) can be obtained on a large scale by the enzymatic process, followed by purification. The variation in the glucose units contained in these CDs allows the generation of cavities of varying sizes within the CD structure. CDs are strongly soluble in water but have a partially hydrophobic cavity capable of forming inclusion complexes with other molecules in an aqueous medium. The steric, functional, and polarity compatibility of the molecules occupying the cavity of CDs limits such inclusion ability (Bragança Carvalho et al., 2019; Morin-Crini et al., 2019; Liu et al., 2020). The high water solubility of CDs makes their application in aqueous remediation treatments impossible. Still, their immobilization in an insoluble matrix can confer relevant gains in the molecule-sequestering properties of the material. CD–silica (CDSi) complexes are insoluble in water, which makes them greatly interesting as adsorbent materials for aqueous pollutants (Morin-Crini et al., 2019; Liu et al., 2020; Gallo et al., 2024). The adsorption of pesticide contaminants on materials based on CDs has been widely studied due to characteristics such as their specific affinity to pollutants, efficiency, and simple operation and the low cost of the materials produced (Li et al., 2018; Wacławek et al., 2022). Poly(vinyl alcohol)-CD nanosponges could remove paraquat (PQ) in aqueous media (Martwong et al., 2021). Zeolites functionalized with β-CD efficiently removed the organophosphate pesticides methyl parathion, omethoate, and acephate (Othman et al., 2022). Cymoxanil and imidacloprid were adsorbed on β-CD and activated carbon composites (Utzeri et al., 2021). Sulfentrazone and quinclorac were adsorbed on graphene oxide and β-CD aerogel (Yao et al., 2024). CD cross-linked with poly(vinyl alcohol) and chitosan was reported to adsorb the herbicide bentazon (Sharma et al., 2023).

CD-based adsorbent materials are normally regenerated with alcohols and/or by adjusting the pH of the medium, depending on the pKa of the pollutant. Improvements in the economic viability of using these adsorbents can be obtained by simplifying the synthesis steps and using low-cost support and functionalization components (Wacławek et al., 2022; Ozelcaglayan and Parker, 2023). Access to safe, uncontaminated drinking water is one of the major problems worldwide (World Health Organization, 2020; United Nations, 2023). Emerging contaminants (ECs) in surface and groundwater basins, such as agrochemicals, have greatly increased in recent years (Food and Agriculture Organization of the United Nations, 2018; Food and Agriculture Organization of the United Nations, 2020; Baigorria and Fraceto, 2022a). Although the use of pesticides is necessary for the safe production of food, their excessive and inappropriate application leads to serious water pollution problems (Food and Agriculture Organization of the United Nations, 2018). Due to the great diversity of existing pesticides and the magnitude of their uses, there are concerns about the risk of human exposure and adverse effects on human health (Merel et al., 2018). For this reason, numerous international organizations and institutions prohibit the use or commercialization of pesticides.

PQ (1,1-dimethyl-4,4-bipyridinium dichloride) is an EC of major concern worldwide. Paraquat is a non-selective, fast-acting, broad-spectrum contact fungicide applied in agriculture, forestry, and household crops to control various broadleaf weeds (Judkins and Wente, 2019; World Health Organization, 2020). Due to its high aqueous solubility, PQ readily bioaccumulates in the environment, mainly in aqueous environmental matrices (Judkins and Wente, 2019). Thus, this fungicide can be consumed by humans, seriously affecting their health. PQ has been detected in different water basins at concentrations ranging from 0.03 to 30 mg.L^−1^ (World Health Organization, 2018; World Health Organization, 2020). Traces of PQ have been found in water bodies in the United States, Japan, China, South Korea, France, Taiwan, Brazil, Argentina, Italy, Germany, and Spain (Veríssimo et al., 2018; Zyoud, 2018; REAB-MdP, 2021). Other relevant pesticides found in surface and groundwater in various countries (United States, Argentina, European Union countries, China, Colombia, Chile, Brazil, and Serbia) are atrazine (ATZ) and carbendazim (CBM) (Agency for Toxic Substances and Disease Registry, 2003; World Health Organization, 2010; World Health Organization, 2018; Pérez et al., 2021; REAB-MdP, 2021). ATZ (6-chloro-N^2^-ethyl-N^4^-isopropyl-1,3,5-triazine-2,4-diamine) is a selective systemic herbicide of the chlorotriazine class, used for the control of broadleaf weeds and some grasses in crops such as maize, sorghum, sugarcane, soybean, and tea (World Health Organization, 2018; SENASA, 2021). ATZ is a pre- and post-emergence herbicide (World Health Organization, 2010).

Moreover, CBM (methyl-1-H-benzimidazole-2-ylcarbamate) is a broad-spectrum systemic fungicide used in pre- and post-harvest stages in sunflower, rice, wheat, citrus, and other crops (Singh et al., 2016). Both pesticides, ATZ and CBM, are banned in the European Union and restricted in other countries such as the United States, Brazil, Uruguay, Oceania, and Africa (Singh et al., 2016; Baigorria and Fraceto, 2022c). The contamination of freshwater basins with these ECs is a problem for society. Obtaining safe water for human consumption and the development of materials and technologies for this purpose are significant challenges for the scientific community. Not all technologies applied in urban aqueous treatment plants can efficiently reduce the concentrations of ECs to levels that do not affect human health because they are ECs. Therefore, adsorption technologies are highly efficient for eliminating these ECs.

Hybrid adsorbent materials such as CDSi composites with novel characteristics and properties for their application in aqueous PQ removal treatment were developed as the main objective of this work. The CDSi composites were synthesized from innovative, green, environmentally friendly, and sustainable methodologies. In addition, the hybrid adsorbent composites were characterized using different physicochemical and morphological techniques, their capacity as adsorbents of aqueous PQ under different experimental conditions, and their behavior against other pesticides such as ATZ and CBM.

## 2 Materials and methods

### 2.1 Materials

α-Cyclodextrin (α-CD), β-cyclodextrin (β-CD), and γ-cyclodextrin (γ-CD) with purity > 98% were purchased from Sigma-Aldrich Co. Silica gel (particle size 5 < 50 µm), hydrochloric acid (HCl), anhydrous citric acid (reagent grade, 99.5%), sodium hydroxide (NaOH), xylene, acetone (ASC grade, ≥99.5%), and absolute ethanol were purchased from Vetec. The PQ pesticide of >98% purity was purchased from Sigma-Aldrich Co. for the aqueous removal tests.

### 2.2 Cyclodextrin–silica nanocomposites

CDSi nanocomposites were previously synthesized according to the methodology proposed by Bragança Carvalho et al. (2019). In brief, silica was previously activated in an acidic medium, washed with water to achieve neutral pH, and dried at 150°C. The adsorbent composites were obtained by refluxing in 50 mL of xylol (at 140°C/6 h), mixing with 2 g of previously activated cyclodextrin and silica and citric acid. The products obtained were consecutively washed in water, acetone, and ethanol. Subsequently, they were dried at 90°C, crushed, and sieved (60 US Mesh).

### 2.3 Characterization assays

The nanocomposites were characterized by N_2_ adsorption analysis, thermogravimetric analysis (TGA), differential thermal analysis (DTA), Fourier-transform infrared (FTIR) spectroscopy, solid-state ^13^C nuclear magnetic resonance (NMR) spectroscopy, and point of zero charge (PZC).

The N_2_ adsorption–desorption technique at 77 K was used to determine the specific surface area and porosity of the CDSi composites (BELSORP-mini II BEL, Japan). The samples were previously degassed at 110°C, and the adsorption isotherms were prepared with ultrapure nitrogen (>99.9995%) by applying a stepwise method over a range of relative nitrogen pressures (up to 0.98). To determine the specific surface area, the Brunauer, Emmett, and Teller (BET) method was used (Brunauer et al., 1938). The t-plot method was used to estimate the micropore volume and external surface area (Barrett et al., 1951; de Boer et al., 1966), while the Barrett, Joyner, and Halenda (BJH) method was used to evaluate mesoporous size distribution (Barrett et al., 1951).

The TGA and DTA curves were obtained by heating samples (∼10 mg, 25°C–600°C) under an N_2_ atmosphere at a heating rate of 10°C min^−1^ and a gas flow rate of 50 mL min^−1^ using Shimadzu equipment with a DTG-60AH detector.

FTIR spectroscopy was carried out on a Shimadzu spectrometer (IRAffinity-1 and Jasco FT/IR) a 410 Fourier Transform Infrared Spectrometer, using a potassium bromide (KBr) tablet. The sample percentage in the KBr tablet was in all cases 2 %w^sample^/w^KBr^. The spectra were obtained from 4,000 to 400 cm^−1^ at a resolution of 8 cm^−1^.

Solid-state cross-polarization magic angle spinning ^13^C NMR experiments (CP/MAS ^13^C NMR) were carried out on a Bruker Avance III 400-MHz Spectrometer (magnetic field of 9.4 T and dual-channel MAS probe of 4.0 mm). Powdered samples were packed in 4-mm ZrO_2_ rotors and sealed with Kel-F hermetic seals (rotation at 12 kHz). ^13^C chemical shifts were reported in parts per million (ppm) referenced externally to adamantane (CH_2_ peak adjusted to 38.5 ppm).

The surface charge of the CD composites was determined by PZC, following the methodology described by Copello et al. (2014). A fixed sample mass (2 mg) was stirred for 24 h in a fixed amount of deionized water. The initial pH (pH_i_) of the solutions was adjusted between 2 and 10 using HCl or NaOH (0.1 M). After stirring the solutions at room temperature, the difference between the measured pH_f_ and pH_i_ (ΔpH) was plotted. pH^PZC^ was obtained from the intersection of each sample with the abscissa axis.

### 2.4 Pollutant water remediation assays

PQ adsorption studies were carried out from batch assays, starting from a 200 mg.L^−1^ PQ stock solution (in water). All assays were performed in triplicate. UV–visible absorption spectroscopy performed using a Varian Cary 50 Scan UV/vis Spectrophotometer was used to quantify PQ. The analytical equation y = (0.06481 ± 0.00004). x + (0.0047 ± 0.0006) (*r* ^*2*^ = 0.99999 −detection limit [DL] = 0.07 mg.L^−1^ −quantification limit [QL] = 0.24 mg.L^−1^) was obtained from the calibration curve. The PQ removal efficiency (*E%*) and the PQ adsorption capacity (*q*) of the CDSi composites were obtained from Equations 1, 2:

$$E\left(\%\right)=\left(\frac{c_{0}-\;c_{t}}{c_{0}}\right)x\;100,$$ (1)

$$q\left(mg.\;g^{-1}\right)=\left(\frac{c_{0}-\;c_{t}}{w_{adsorbent}}\right)x\;V,$$ (2)

where *c*_*o*_ and *c*_*t*_ (mg.L^−1^) are the initial PQ concentration and PQ concentration at time *t*, respectively; *w*_*adsorbent*_ (g) is the mass of adsorbent used; and *V* (L) is the volume of the PQ solution (Baigorria and Fraceto, 2022b).

#### 2.4.1 Concentration variation effects

The effects of varying adsorbent concentrations (α-CDSi, β-CDSi, and γ-CDSi—0.02 < %w/v < 0.15) on PQ removal treatments (20 mg.L^−1^) were explored. The influence of varying contaminant concentrations (4 mg.L^−1^ < [PQ] < 25 mg.L^−1^) on adsorption treatments was also analyzed using a fixed concentration of each adsorbent CDSi composite (0.02 %w/v). All analyses were carried out for 24 h at room temperature (25°C), with the pH-controlled (8 ≤ pH ≤ 10).

#### 2.4.2 Temperature and pH effects

The effect of pH on the PQ removal treatment (20 mg.L^−1^) was analyzed using α-CDSi, β-CDSi, and γ-CDSi as adsorbents (0.02 %w/v). The pH adjustment was performed by adding 0.1 M solutions of HCl or NaOH as appropriate to obtain a pH range of 2 ≤ pH ≤ 10 (t_remotion_ 24 h, 25°C). Furthermore, PQ adsorption analyses (20 mg.L^−1^, CDSi_concentration_ = 0.02 %w/v, 8 ≤ pH ≤ 10, 24 h) were performed at different temperatures (25°C ≤ T° ≤ 45°C) to analyze the effect of temperature on the adsorption treatments.

#### 2.4.3 Contact time effect

The effects of the contact time of the adsorbent materials on PQ solutions at a concentration of 20 mg.L^−1^ of the contaminant were studied. The tests were conducted at a pH range of 8 ≤ pH ≤ 10, using the CDSi complex at a concentration of 0.02% w/v at room temperature.

#### 2.4.4 Analysis of the adsorption mechanism

Based on the data obtained in Section 2.4, different models were applied to estimate the type of mechanism (physical or chemical) by which PQ adsorption occurs in the CDSi composite.

The adsorption process was studied by applying Freundlich and Langmuir isotherm models (Langmuir, 1997). Equations 3, 4 describe the Freundlich and Langmuir models, respectively.

$$q_{e\;}=k_{F}.\;c_{e}^{\frac{1}{n}},$$ (3)

$$q_{e}=\frac{Q_{0}\;.\;{\;k}_{L\;}.\;c_{e}}{1+{\;k}_{L\;}.\;c_{e}},$$ (4)

where *q*_*e*_ (mg.g^−1^) is the amount of PQ adsorbed at equilibrium, *k*_*F*_ [(mg^(1/n)^.L^n^).g^−1^] is the Freundlich constant, *c*_*e*_ (mg.L^−1^) is the pollutant concentration at equilibrium, *1/n* is the heterogeneity factor, *Q*_*0*_ (mg.g^−1^) is the maximum adsorption capacity, and *k*_*L*_ (L.mg^−1^) is the Langmuir equilibrium constant (Langmuir, 1997; García-Carvajal et al., 2019; Chu, 2021).

According to the Freundlich model, the energy distribution in the adsorbent sites is not uniform, i.e., there is heterogeneity in the adsorption sites. In contrast to the previous model, the Langmuir model implies that the adsorption process is homogeneous at the adsorption sites due to their energetic uniformity (Langmuir, 1997; García-Carvajal et al., 2019).

Thermodynamic parameters associated with different energy changes through the adsorption process at various temperatures were determined from the PQ removal tests. The variations in the entropy [*∆S* (kJ.mol^−1^. K^−1^)], enthalpy [*∆H* (kJ.mol^−1^)], and Gibbs free energy [*∆G* (kJ.mol^−1^)] of the process were determined from Equations 5, 6 and the van’t Hoff plot (Chen et al., 2011; Dawood and Sen, 2012; Oladipo and Gazi, 2014; Foroutan et al., 2021).

$$\mathrm{Ln}\;\frac{q_{e}}{c_{e}}=\frac{∆S}{2.303\;R}-\frac{∆H}{2.303\;R.T},$$ (5)

∆G=∆H−T.∆S, (6)

where *c*_*e*_ (mg.L^−1^) is the pollutant concentration at equilibrium, *q*_*e*_ (mg.g^−1^) is the amount of PQ adsorbed at equilibrium, *R* (8.3124 J. K^–1^.mol^−1^) is the gas constant, and *T* (K) is the temperature (Chen et al., 2011; Dawood and Sen, 2012; Oladipo and Gazi, 2014).

The interactions between the adsorbent material and the contaminants can be estimated by analyzing *∆S*, *∆H*, and *∆G* values. The Gibbs free energy, in addition to indicating the spontaneity (*∆G* < 0) or non-spontaneity (*∆G* > 0) of the process, provides clues to the nature of the adsorption mechanism. Physisorption mechanisms exhibit energies of −20 kJ .mol^−1^ < *ΔG* < 0 kJ .mol^−1^, while energies of −80 kJ .mol^−1^ < Δ*G* < −400 kJ .mol^−1^ indicate the existence of chemisorption during the process. The adsorption process using these materials may be chemisorption for those processes where ΔG > 0, exceeding the mentioned ranges (Oladipo and Gazi, 2014; Baigorria and Fraceto, 2022b).

Adsorption kinetic studies were analyzed using the Elovich, pseudo-first-order (PFO), and pseudo-second-order (PSO) models. Equations 7–9 describe the Elovich, PFO, and PSO models, respectively (Elovich and Larinov, 1962; Oladipo et al., 2019; Wang and Guo, 2020):

$$q_{t}=\frac{1}{\alpha }.\;\ln\left(1+\alpha \beta t\right),$$ (7)

$$q_{t}=q_{e}\;.\;\left(1-\;e^{-k_{1}t}\right),$$ (8)

$$q_{t}=\frac{q_{e}^{2}\;.\;k_{2\;}.\;t}{1+k_{2}\;.\;q_{e}\;.\;t\;},$$ (9)

where *q*_*e*_ (mg.g^−1^) is the adsorption capacity of the materials at equilibrium, *q*_*t*_ (mg.g^−1^) is the adsorption capacity of the materials at time *t*, *α* (mg.g^−1^.min^−1^) is the initial adsorption rate, *β* (g.min^−1^) is the desorption constant, *k*_*1*_ (min^–1^) is the equilibrium constant of the PFO model, and *k*_*2*_ (g.mg^−1^.min^−1^) is the equilibrium constant of the PSO model (Elovich and Larinov, 1962; Oladipo et al., 2019; Wang and Guo, 2020).

All the kinetic models analyze various aspects of the process, which generally involves three stages: external and internal diffusion and adsorption of the adsorbate molecules on the active sites of the adsorbent. The empirical Elovich model assumes that both the interactions between the adsorbed molecules and the desorption process substantially affect the adsorption process that occurs with minute surface coverage. Furthermore, it assumes that surface heterogeneity in the adsorbent structure mainly involves the chemical adsorption phenomenon (Elovich and Larinov, 1962). The PFO empirical model describes the diffusional, or mass transfer, stage of adsorbate molecules to the active sites of the adsorbent (Wang and Guo, 2020), while the PSO empirical model describes the adsorption process on the active sites themselves (Wang and Guo, 2020).

#### 2.4.5 Complementary analysis: other pollutants

A supplementary analysis was performed using other agrochemicals of different chemical structures: CBM and ATZ. Starting solutions of 200 mg.L^−1^ of ATZ (in methanol) and CBM (in water) were used. Removal assays were carried out for 24 h at a pH range of 8 ≤ pH ≤ 10 using a 0.02% w/v concentration of the CDSi composite at room temperature.

ATZ and CBM were quantified on Thermo Scientific UltiMate 3000/RS equipment using high-performance liquid chromatography (HPLC). The samples to be quantified were filtered using a syringe filter (PTFE FI 13 mm, 0.22 μm; Allcrom) before analysis. A Gemini RP-18 Column (5 μm, 150 mm × 4.6 mm; Phenomenex) with an elution gradient of 39% acetonitrile and 0.5% methanol and a column temperature of 25°C (a flow rate of 1.0 mL.min^−1^ and a detector wavelength of 288 nm) was used for CBM determination. The analytical equation obtained for CBM was y = 5.67.x + 0.56 (r^2^ = 0.99999, DL = 0.004 μg.L^−1^, and QL = 0.0146 µg.L^−1^) (Baigorria and Fraceto, 2022c). An elution gradient of 50% acetonitrile on a Luna C18 Column (5 μm, 100 Ả, 250 × 4.6 mm, flow rate 1.5 mL.min^−1^, column temperature 30°C, and detector wavelength 223 nm; Phenomenex) was used for ATZ quantification (Bragança Carvalho et al., 2023). The analytical equation obtained for ATZ was y = 10.23.x + 0.92 (r^2^ = 0.99774, DL = 0.254 μg.L^−1^, and QL = 0.847 µg.L^−1^).

### 2.5 Statistical analysis

Statistical analysis of the data was performed by statistical methods such as one-way variance using Origin 8 Pro software. All results were reported together with the corresponding standard deviation.

## 3 Results and discussion

### 3.1 Characterization studies

The functionalization of silica with cyclodextrins was reported in previous works (Bragança Carvalho and de Matos Alves Pinto, 2012; Bragança Carvalho et al., 2014; Baracho et al., 2015). However, this section presents results from the optimization of the synthesis process and a robust molecular characterization of the surface characteristics and composition of the synthesized materials. The spectroscopy analyses and the thermals for the β-CDSi composite were previously presented by Bragança Carvalho et al. (2019); therefore, in this paper, we present these characterizations only for the α-CDSi and γ-CDSi composites.

**FIGURE 1:**
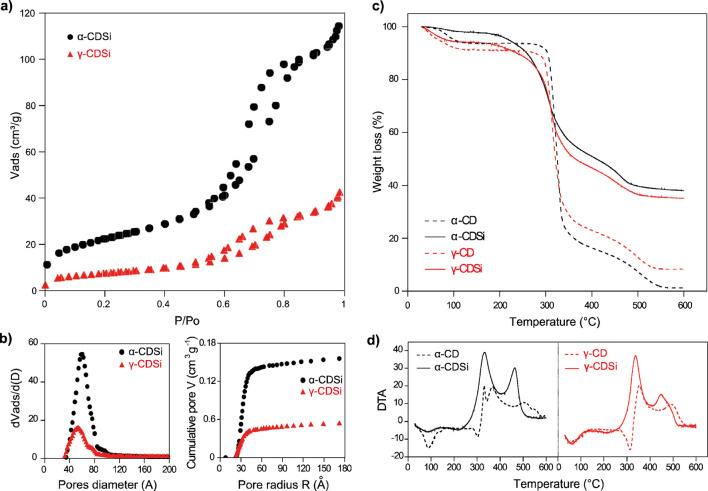
**(A)** N_2_ adsorption–desorption isotherms at 77 K and **(B)** mesopore size distribution by the BJH method and accumulated mesopore volume for the α-CDSi and γ-CDSi composites. **(C)** Thermogravimetric curves and **(D)** DTA for samples of α-CD, γ-CD, and the respective composites.

The N_2_ adsorption–desorption isotherms (Figure 1A) for both composites showed a hysteresis loop typical for mesoporous materials (Thommes et al., 2015). The surface areas decrease with the coating of the silica, inverse to the size of the functionalized oligosaccharide, with specific values of 80.0 ± 6.6 m^2^.g^−1^ and 26.5 ± 6.4 m^2^.g^−1^ for α-CDSi and γ-CDSi, respectively. In a previous study, the surface area value determined for the β-CDSi composite was intermediate to the CDs described in the present study (Bragança Carvalho et al., 2019). The mesopore size distribution (Figure 1B) showed a similar diameter between the composites, approximately 4–8 nm, and a mesopore volume of 0.17 and 0.06 m^3^.g^−1^ for α-CDSi and γ-CDSi, respectively. For β-CDSi, the reported mesopore volume was 0.14 m^3^.g^−1^, demonstrating that the blockage for N_2_ access to external surfaces is more significant for CDs with larger diameters, which must occupy or block the mesopores in a more effective way for the diffusion of the adsorbate to the adsorption sites existing on the silica surface, and/or they can be functionalized to the silica by a greater number of binding agents, which would consequently occupy more adsorption sites. Although there is a reduction in the surface area of the silica with the functionalization (Bragança Carvalho et al., 2019) and also with the size of the anchored CD, the stability of the energy constant C (110–128) for the composites and the inorganic matrix indicates that in an aqueous medium, factors such as hydrophobicity and functionalization density should control the adsorption properties of the matrices.

The mass loss curves (Figure 1C), obtained by TGA/DTA, report changes in the enthalpies and the thermogravimetric profile of the synthesized materials, highlighting physicochemical changes in CDs anchored to silica. The initial mass losses, up to 130°C, refer to the endothermic process of sample dehydration (Figure 1D). While α-CDSi presented a degree of hydration of 1.3%, for γ-CDSi, the percentage was 5.9%. The lower degree of hydration for the α-CDSi composite may be associated with CD becoming more amorphous with functionalization than γ-CD as water molecules are also related to the stabilization of the crystalline structure of CDs (Szejtli, 1988). The absence of the endothermic peak at approximately 300°C, associated with the melting temperatures of the CDs, and the persistence of two exothermic peaks, similar to the thermal degradation profiles of the respective unanchored CDs, attest to the functionalization of silica by CDs.

TGA allows estimating the relative composition of the new materials, in which the percentage of anchoring of CDs in silica was 60.6% and 58.9% for α-CDSi and γ-CDSi, respectively. This value is significantly higher than 41.0% (α-CDSi) and 47.0% (γ-CDSi) reported by Baracho et al. (2015). The increase in reaction yield highlights the importance of acid treatment for activating the silica surface, making more silanol groups available for functionalization bonds (Jiang et al., 2006; Bragança Carvalho et al., 2019). In this way, optimization in the synthesis process should provide the new material with operational gains in applications in adsorption systems, increasing its ability to interact with other molecules.

**FIGURE 2:**
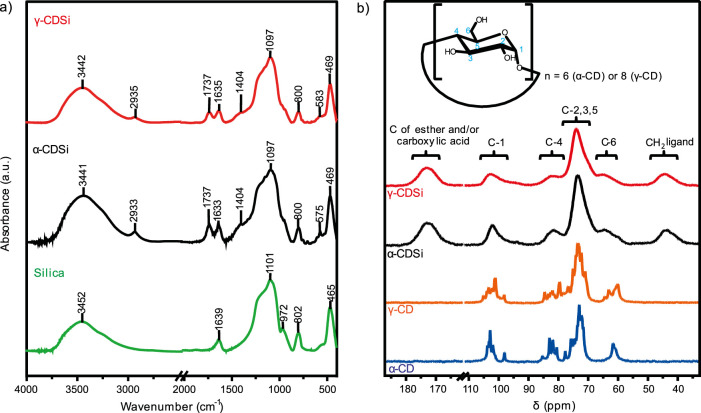
**(A)** Infrared spectra of the silica, α-CDSi, and γ-CDSi samples. **(B)** ^13^C NMR spectra (δ/ppm) for non-functionalized cyclodextrins and their respective silica composites (α-CDSi and γ-CDSi).

Just to clarify, what is being said is that using the infrared spectrum it is possible to correctly observe the incorporation of CD into the silica surface (Figure 2A). The intensity variation and the shifts in the characteristic absorption bands of the compounds concerning their precursors make it possible to observe this effect. An increase in intensity and change in the profile of the absorption band of the α-CDSi and γ-CDSi composites are observed at approximately 3,400 cm^−1^, concerning pure silica. These bands are characteristic of the O-H stretching of the silanol groups and adsorbed water molecules and begin to be contributed by the O-H stretching of the CDs. In the composites, the characteristic C-H axial deformation band of cyclodextrin is noted, slightly shifted to a higher wavelength of 2,927 cm^−1^, and the band at 1,737 cm^-1^ refers to the carbonyl of the binding agent used for functionalization. The functionalization affects the angular vibrations of the water molecules, possibly due to the hindrance of some vibrational modes of the new structure, causing a change in intensity and shift in the characteristic band to shorter wavelengths, approximately 1,635 cm^−1^ (Bragança Carvalho et al., 2019). The bands attributed to the deformation of free silanol groups and Si-OH stretching at 972 and 802 cm^−1^, respectively (Trofymchuk et al., 2017), also show reduced intensity, possibly due to their use as reactive centers in functionalization bonds with CDs.

The ^13^C NMR spectra in the solid state, shown in Figure 2B, and the respective assignments for the constituent carbon signals, shown in Supplementary Table S1, corroborate the other characterization techniques to confirm the incorporation of CDs onto the silica surface. The functionalization process caused an impediment to the molecular movements of the CDs, resulting in an abrupt reduction in the definition of the respective signals when compared to the free forms. Comparing the mean of the specific signals by each carbon type with the centered displacement values of the respective broadened peaks (Supplementary Table S1) showed that the largest variations were 3.20 and 1.76 in C-6 for α-CD and γ-CD, respectively. The spectra of the composites also show characteristic signals of CH_2_ at 44 ppm from the citric acid structure and carbonyl groups associated with ester-type bonds and carboxylic acids at 173 ppm. Thus, functionalization occurs through an esterification reaction by the hydroxyl linked to the C-6 of the CDs with a carboxylic group from citric acid and silanol, according to the reaction mechanism reported for the β-CDSi composite (Bragança Carvalho et al., 2019).

The superficial charge distribution in the CDSi composites was estimated from the PZC measurements. pH^PZC^ values of 6.12, 5.33, and 4.67 were obtained for the composites α-CDSi, β-CDSi, and γ-CDSi, respectively (Supplementary Figure S1a). This characterization test allows us to analyze the PQ adsorption processes in later sections.

### 3.2 PQ adsorption studies

**FIGURE 3:**
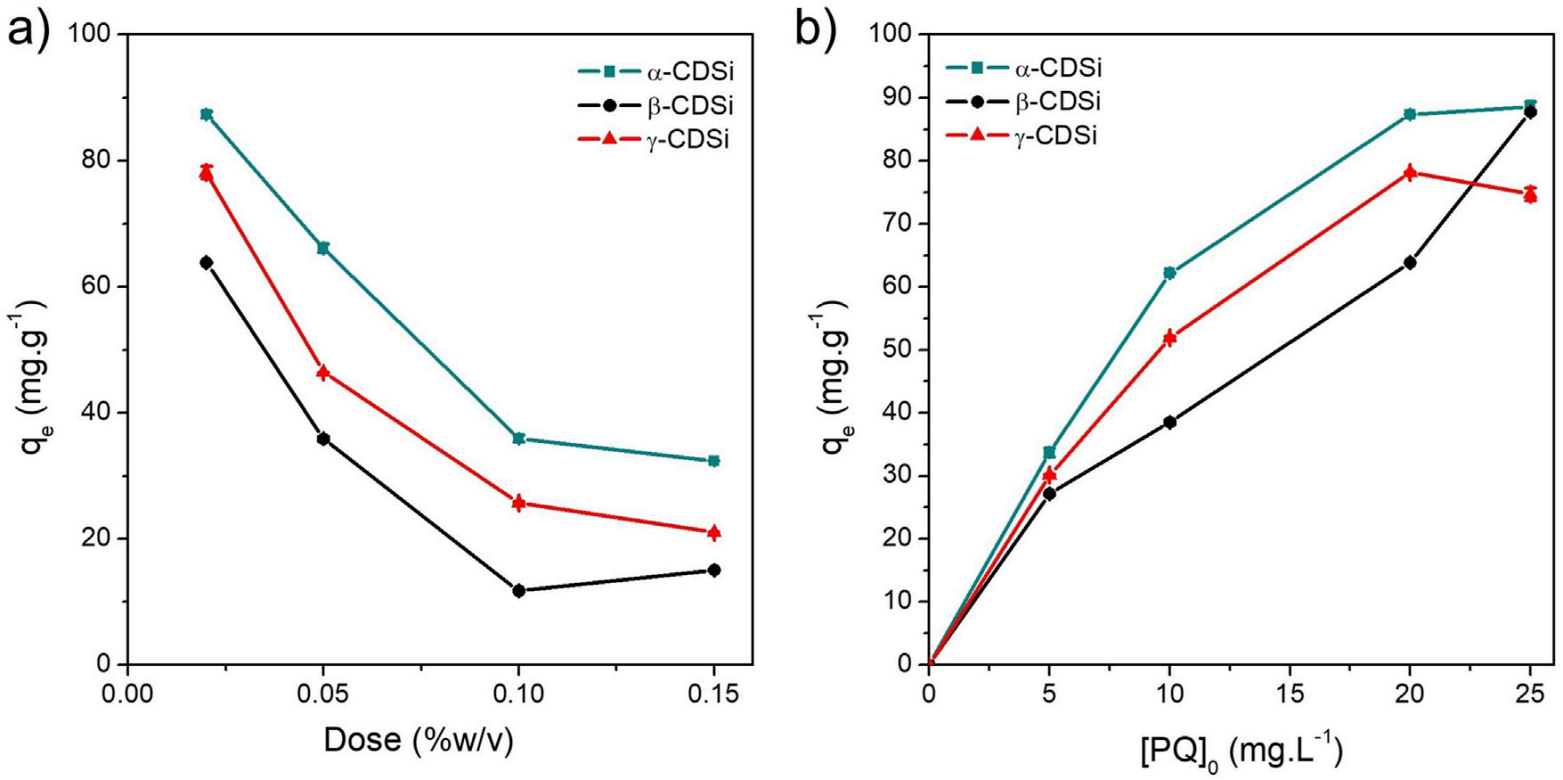
**(A)** Effect of the adsorbent dose (0.02, 0.05, 0.10, and 0.15 %w/v) on the PQ removal treatment ([PQ]_0_ = 20 mg.L^−1^, 25°C). **(B)** Effect of the initial PQ concentration (5, 10, 20, and 25 mg.L^−1^) on the PQ removal treatment (adsorbent dose = 0.02 %w/v, 25°C, 8 ≤ pH ≤ 10).

PQ removal treatment studies using CDSi composites as adsorbents analyzed different aspects that affect the adsorption process. First, the effect of increasing the adsorbent dose material on the remediation treatment was analyzed. Figure 3A shows that as the CDSi dose increases, the amount of PQ (mg.g^−1^) removed decreases. The behavior observed is attributed to the interactions between the PQ molecules and the surface and active sites of the adsorbent material (Arias et al., 2009; Rizzi et al., 2020). A higher adsorbent dosage increases the number of available sites, so the unsaturation of these sites decreases *q*_*e*_ (mg.g^−1^) (Oladipo and Gazi, 2014; Rizzi et al., 2020). Several authors attribute this effect to the possibility that at low adsorbent doses, there is a greater number of interactions between the pollutant and the material, coupled with a small desorption effect, unlike when the adsorbent dose is higher (Ennajih et al., 2012; Rizzi et al., 2020).

Moreover, under these experimental conditions, it was observed that α-CDSi presented a higher adsorption capacity than γ-CDSi and β-CDSi. Maximum *q*_*e*_ values of 87.36, 78.19, and 63.88 mg.g^−1^ were obtained for the composites α-CDSi, γ-CDSi, and β-CDSi, respectively, for adsorbent doses of 0.02 %w/v. Figure 3B shows the PQ concentration effect on each removal treatment when the lowest dose of CDSi composites (0.02 %w/v) was used. The removal capacity of CDSi composites increased as the initial dose of PQ increased. Under the experimental condition where the initial PQ concentration was 25 mg.g^−1^, the highest values of *q*_*e*_ were obtained, being 88.58, 87.79, and 74.74 mg.g^−1^ for α-CDSi, β-CDSi, and γ-CDSi, respectively. Analyzing the relationship between the CDSi dose and PQ dose showed that by reducing this ratio, a greater number of active adsorption sites are available in the CDSi composites (Rizzi et al., 2020). This is attributable to the phenomenon of mass transfer of PQ from the contaminant solution to the active sites of the adsorbent material (Oladipo and Gazi, 2014; Rizzi et al., 2020).

According to the results, α-CDSi and γ-CDSi performed better in removing PQ from water than β-CDSi. Studies reported that aqueous adsorption treatments of methylene blue exhibited the same trend in the removal results for the α-CDSi, β-CDSi, and γ-CDSi composites (Bragança Carvalho et al., 2014; Bragança Carvalho et al., 2022). The equilibrium adsorption capacities for the cationic dye methylene blue and the pesticide PQ^2+^ were higher for the α-CDSi and γ-CDSi, while β-CDSi showed lower contaminant-adsorption capacities. This behavior is attributed to the structural difference between the CDSi composites studied. The chemical structure of the composites α-CDSi and γ-CDSi presents high similarity. There is only a difference in the number of glucose monomers in their structures, i.e., six for α-CDSi and eight for γ-CDSi (Bragança Carvalho et al., 2022). In addition, although the complex β-CDSi possesses seven glucose monomers, it has a higher structural rigidity caused by the existing H-bonding between the oxygens of its glycosidic structure (Cai et al., 2008; Bragança Carvalho et al., 2014; Bragança Carvalho et al., 2022). The structural difference of the complexes directly affects the adsorption process by decreasing the diffusion/transport rate of the PQ molecules (adsorbate) toward the active sites of the adsorbent materials.

Equilibrium adsorption capacity (*q*_*e*_) variation was analyzed under different thermal conditions with fixed doses of adsorbents and PQ. An increase in the temperature of the PQ removal treatments resulted in a decrease in *q*_*e*_ for the CDSi composites (Supplementary Figure S1B). Room temperature (25°C) was the most suitable for the aqueous PQ adsorption treatment. Values of *q*_*e*_ of 87.36, 63.88, and 78.19 mg.g^−1^ were obtained for α-CDSi, β-CDSi, and γ-CDSi, respectively. These results indicate exothermic-type adsorption processes (Oladipo and Gazi, 2014; Baigorria and Fraceto, 2022b). Studies of the pH variation effect on the removal treatments showed that as the pH of the solutions increased, the CDSi composite adsorption capacity increased considerably. Supplementary Figure S1B shows that for pH > 8 for the composites α-CDSi and γ-CDSi, *q*_*e*_ did not vary, showing a plateau in the graph. These results highlight the influence of the surface charge variation in the CDSi adsorbent composite on the interactions with the PQ molecules. PQ is produced in the chloride salt form of 1,1′-dimethyl-4,4′-bipyridylium (Supplementary Figure S2). Two positive charges are distributed on the aromatic rings in its dichloride salt structure (Aguiar et al., 2023). At near-pH values where the surface charge of the CDSi composites is positive (pH < pH^PZC^), the interactions with the PQ^2+^ molecules are lower. Meanwhile, at pH > pH^PZC^, the surface charge of the adsorbent materials is negative, thus favoring interactions with PQ^2+^ molecules.

**FIGURE 4:**
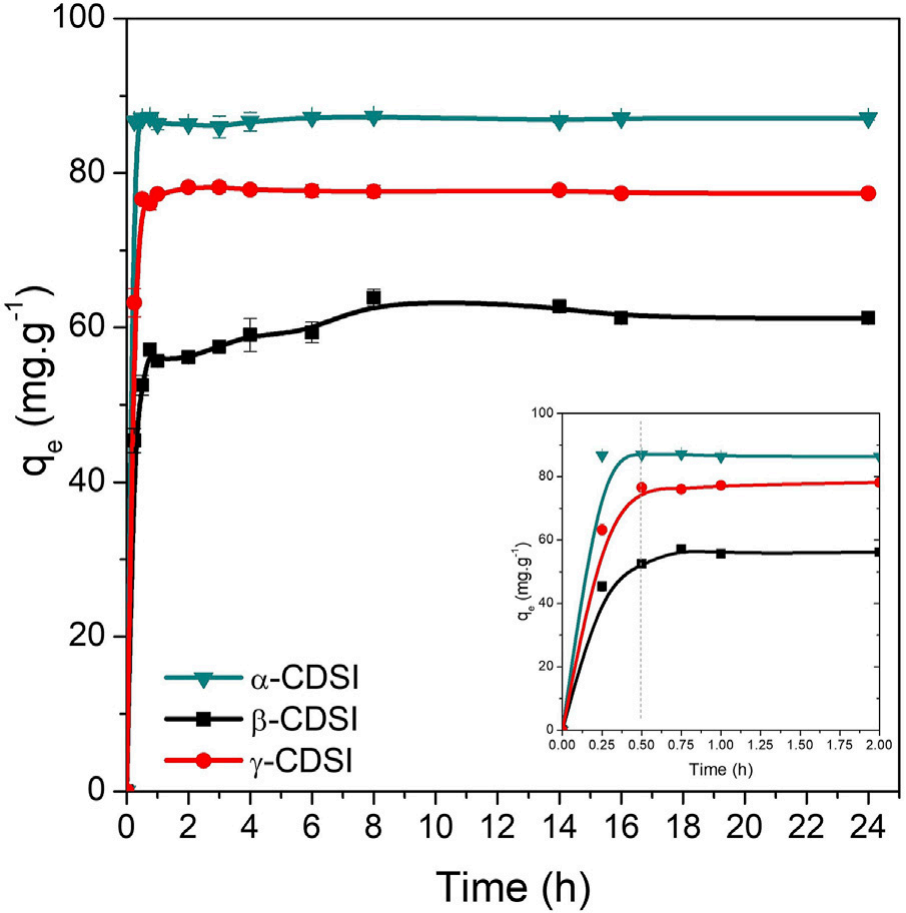
Time effect on the PQ adsorption treatment ([PQ]_0_ = 20 mg.L^−1^, adsorbent dose = 0.02 %w/v, room temperature).

Mass transfer from a liquid-phase adsorbate to the surface of a solid material is a process known as adsorption (Wang and Guo, 2020). The influence of time analyzed in this study provides information about the adsorption rate, mechanisms involved, and treatment performance, among others. Three stages are evidenced during the mass transfer process studied over time (Zeng et al., 2019; Wang and Guo, 2020). The first step of the process involves external diffusion of the adsorbate to the adsorbent external surface. Next is the second stage of internal diffusion, in which the adsorbate molecules diffuse toward the active sites of the material. Finally, the third step is the complete adsorption of the adsorbent molecules onto the active sites of the adsorbent material (Zeng et al., 2019; Wang and Guo, 2020). Figure 4 shows the time-dependent behavior of the PQ adsorption process on CDSi composites. A high PQ-removal rate at initial treatment times is observed. Removal rates of 86.75, 45.38, and 63.23 mg.g^−1^ were obtained for α-CDSi, β-CDSi, and γ-CDSi, respectively, within only 15 min of the start of the PQ removal treatment. A small increase in *q* was observed at 0.5 h of treatment for the α-CDSi (87.08 mg.g^−1^) and γ-CDSi (63.23 mg.g^−1^) composites, values that remained stable throughout the treatment. The composite β-CDSi showed stability of *q* from 1 h of treatment. These results indicate that the diffusion stages (external and internal) in the process of mass transfer of PQ to the surface and active sites of α-CDSi and γ-CDSi were at a high rate of speed, whereas for β-CDSi, the rate of velocity was slightly lower. Equilibrium *q* (*q*_*e*_) was observed for all the CDSi composites studied for periods exceeding 1 h, showing a constant adsorption rate without desorption or any undesired effect at longer treatment times. The difference in the results obtained for α-CDSi, β-CDSi, and γ-CDSi composites is highly influenced by the structural differences of the composites, as explained previously.

#### 3.2.1 Analysis of adsorption mechanisms

An appropriate understanding of an adsorption process involves two basic aspects: the adsorption equilibrium and the kinetics of the process. Analysis of the isothermal and thermodynamic models provides information about the final state of a system. Meanwhile, the study of the kinetic process indicates the variations that occur during the adsorption process (Azizian, 2004).

Isothermal studies of the adsorption process show a plausible adsorbed molecule distribution between the solid and liquid phases during the equilibrium adsorption process. The empirical Langmuir and Freundlich isotherm models were applied to study PQ adsorption on the composites α-CDSi, β-CDSi, and γ-CDSi. Supplementary Figure S3 shows the nonlinear fit of the experimental data obtained with these models (Equations 3, 4). As mentioned above, the parameters obtained from the fit are shown in Table 1. PQ adsorption on the materials α-CDSi and γ-CDSi was consistent with both empirical models studied. This indicates the contribution of physisorption and chemisorption mechanisms during the adsorption process of these materials, whereas the experimental data on PQ adsorption on β-CDSi did not show a considered fit with any of the studied models.

**TABLE 1:** Isotherm parameters for PQ adsorption data by α-CDSi, β-CDSi, and γ-CDSi.

Sample	Langmuir			Freundlich		
	Q_0_	k_L_	r^2^	k_F_	1/n	r^2^
	(mg.g^-1^)	(L.mg^-1^)		((mg^(1/n)^.L^n^)/g)		
α-CDSi	116.07	0.643	0.9934	45.81	0.409	0.9117
β-CDSi	257.91	0.053	0.9617	15.90	0.756	0.9364
γ-CDSi	94.47	4.801	0.9887	80.19	0.3663	0.8957

The Langmuir isotherm model simulates a PQ adsorption process on the monolayer of CDSi composites (Wang and Guo, 2020). It considers the active sites to be energetically uniform. The Freundlich empirical model, on the other hand, considers that the adsorbent surface is heterogeneous and has mono-multilayer coverage (Wang and Guo, 2020). Considering the parameters obtained from the nonlinear fit of both models, it is possible to obtain information about the mechanism of PQ adsorption on CDSi composites. The adsorption process is an equilibrium process that we can simulate as

$$PQ+{CDSi}_{free\;}\;↔\;{CDSi-PQ}_{ads},$$

where we initially find the adsorbate molecules (PQ) and the adsorbate active sites unoccupied (*CDSi*_*free*_) and, after the process, the occupation of the active sites by the PQ molecules (*CDSi-PQ*_*ads*_). The k_L_ value indicates the affinity of the adsorbate for the active sites of the adsorbent (Azizian et al., 2018). Several authors mention *k*_*L*_ as the adsorption equilibrium constant (Azizian et al., 2018; Wang and Guo, 2020). The *k*_*L*_ value indicates how favored the adsorption process has been. The adsorption process of PQ on β-CDSi evidenced a small *k*_*L*_ value (Table 1). This indicates the existence of numerous free (unoccupied) active sites in the β-CDSi complex. The results were evidenced in the removal tests, where the values of *q*_*t*_ and *q*_*e*_ for PQ adsorption on β-CDSi were substantially lower than those on the adsorbents α-CDSi and γ-CDSi (Figure 4). The value obtained for *k*_*L*_ from the nonlinear fit of α-CDSi was close to 1. Hence, this result indicates only a small difference between the free active sites and those occupied by PQ molecules.

On the other hand, γ-CDSi evidenced a value of *k*_*L*_ >> 1. High *k*_*L*_ values indicate that PQ molecules possibly occupy a large part of the active sites of γ-CDSi. These results agree with the values of *q*_*t*_ and *q*_*e*_ obtained for α-CDSi and γ-CDSi (Figure 4).

The Freundlich isotherm model equation (Equation 3) allows a nonlinear experimental data fit. The “*n*” parameters obtained indicate the degree of intensity of PQ adsorption on CDSi complexes (Rivas et al., 2014). Values of *n* of 2.44, 1.32, and 2.75 were observed for α-CDSi, β-CDSi, and γ-CDSi, respectively. Small values of *n* indicate greater heterogeneity in the active sites of the material. That is, β-CDSi possesses a surface with higher heterogeneity than α-CDSi and γ-CDSi (Rivas et al., 2014; Wang and Guo, 2020).

Moreover, since the *n* value obtained for the PQ adsorption process on β-CDSi is close to 1, it indicates a highly disfavored adsorption process (Romita et al., 2019). The values of *n* obtained for α-CDSi and γ-CDSi indicate that the PQ adsorption process is favored to a greater degree than in β-CDSi (Romita et al., 2019). Langmuir and Freundlich empirical models possess higher reliability or validity only at concentrations above which the fit is nonlinear (Singh, 2016). As seen in Figure 3B, the variation in the experimental data of PQ adsorption on β-CDSi exhibits a nearly linear growth. Therefore, this process does not fit the studied models. The complexes α-CDSi and γ-CDSi show that as *c*_*e*_ increases, the increase of *q*_*e*_ tends to plateau.

The above analysis agrees with the results discussed in Section 3.2. Low and unfavorable PQ adsorption manifested by the β-CDSi composite is due to the existence of free active sites, while for α-CDSi and γ-CDSi, the PQ adsorption performance is higher due to an increase in the occupancy of the active sites of these materials. Appreciable differences attributed to the structural difference between the CDSi composites studied.

Variations in temperature in tests for PQ removal allowed the calculation of thermodynamic parameters involved in the energetic changes during the sorption process (Table 2). The enthalpy variation in the PQ adsorption processes studied indicated processes of an exothermic nature (*ΔH* < 0). On the other hand, the variation in Gibbs free energy (*ΔG*) indicated the spontaneity of the PQ adsorption processes in the studied CDSi composites. Values of −27 kJ.mol^−1^ < *ΔG* < 0 kJ.mol^−1^ were observed (Table 2). This range of *ΔG* values indicates adsorption processes with physisorption mechanisms (Dawood and Sen, 2012; Oladipo and Gazi, 2014; Baigorria and Fraceto, 2022c). In all the cases studied, increased *ΔG* (more positive *ΔG* values) was observed as the temperature increased. This result indicates that, at higher temperatures, the adsorption process of PQ on the active sites of CDSi composites is disfavored (Dawood and Sen, 2012; Oladipo and Gazi, 2014; Baigorria et al., 2023). In addition, thermodynamic studies exhibited small negative process entropy variation (*ΔS*) values. Internal changes in the active sites of the adsorbent materials with little incidence in the adsorption process are those that evidence *ΔS* < 0 (Adelaja et al., 2019; Kennedy et al., 2021).

**TABLE 2:** Thermodynamic parameters for PQ adsorption data by α-CDSi, β-CDSi, and γ-CDSi.

Sample	ΔS	ΔH	ΔG (kJ.mol^−1^)		
	kJ.mol^-^.K^−1^	kJ.mol^-1^	298 K	308 K	318 K
α-CDSi	−0.23	−86.58	−18.04	−15.74	−13.44
β-CDSi	−0.32	−107.61	−11.95	−8.74	−5.53
γ-CDSi	−0.16	−75.47	−26.59	−24.95	−23.31

The adsorption process is strongly time-dependent and involves equilibrium between adsorption and desorption rates of the adsorbate on the active sites of the adsorbent. The influence of time on the adsorption process of PQ on CDSi composites was studied by fitting data with different theoretical kinetic models. Nonlinear fitting of PFO, PSO, and Elovich models was used (Supplementary Figure S3). The theoretical kinetic models studied consider the diffusional processes without considering external factors that disturb the process. Based on the PFO model, it is possible to have indications of the first step of the adsorption process. In this first stage, the diffusion of PQ molecules toward the active sites of the adsorbent materials is generated. Table 3 shows the parameters obtained after the nonlinear fit with the kinetic model.

**TABLE 3:** Kinetic parameters of the PFO, PSO, and Elovich models.

Model	Parameter	α-CDSi	β-CDSi	γ-CDSi
Experimental data	*q*_*e*_^*exp*^ *(mg.g* ^−*1*^ *)*	87.36	63.88	78.19
PFO	*q*_*e*_ *(mg.g* ^−*1*^ *)*	86.84	59.46	77.70
	Δ*q*_*e*_ *(mg.g* ^−*1*^ *)* ^a^	0.52	4.41	0.49
	*k*_*1*_ *(min* ^−*1*^ *)*	0.458	0.087	0.113
	*r* ^*2*^	0.9996	0.9754	0.9990
PSO	*q*_*e*_ *(mg.g* ^−*1*^ *)*	86.84	57.67	76.17
	Δ*q*_*e*_ *(mg.g* ^−*1*^ *)* ^a^	0.52	6.21	1.92
	*k*_*2*_ *(mg.g* ^*-1*^ *.min* ^−*1*^ *)*	1.15	−5.06 × 10^45^	7.68 × 10^39^
	*r* ^*2*^	0.9996	0.9023	0.9628
Elovich	Δ*q*_*e*_ *(mg.g* ^−*1*^ *)* ^a^	1.18	0.09	1.52
	α *(mg.g* ^*-1*^.*min* ^−*1*^ *)*	6.15 × 10^4^	2.82 × 10^6^	8.10 × 10^17^
	β *(g.min* ^−*1*^ *)*	1.22	0.32	0.60
	*r* ^*2*^	0.9976	0.9808	0.9756

^a^ Δq_e_ (mg.g−1) = ǀq_e_^exp^ −q_e_^kinetic model^ǀ.

The value of *k*_*1*_ obtained indicates how fast equilibrium is reached in such an adsorption process. Higher *k*_*1*_ values were observed for α-CDSi, γ-CDSi, and β-CDSi. According to the PFO model, the PQ adsorption process on the α-CDSi and γ-CDSi composites reaches equilibrium faster than that on the β-CDSi composite. Another model studied was the PSO model, which analyzes a second phase of the adsorption process, where the PQ is adsorbed on the active sites of the adsorbent materials. Based on this nonlinear fit, the value of the parameter *k*_*2*_ describing the rate of the adsorption equilibrium is obtained. High *k*_*2*_ values indicate that the adsorption equilibrium is reached relatively quickly. Higher *k*_*2*_ values were observed for the γ-CDSi, indicating that the equilibrium is reached faster than for the other materials studied. These results are shown in Figure 4.

On the other hand, the parameter values obtained in the fit of the experimental data of PQ adsorption on β-CDSi were not consistent. As explained earlier in this section, the adsorption of PQ on β-CDSi was highly disfavored, and the fit with the PSO model was not achieved. Many authors claim that the fit of experimental data with the PSO model does not apply to energetically heterogeneous surfaces, as is the case for β-CDSi (Azizian, 2004; Plazinski et al., 2009).

The Elovich model was also studied in the nonlinear fitting of the obtained experimental data. The Elovich model assumes that the adsorbent material has a highly heterogeneous surface (Elovich and Larinov, 1962; Plazinski et al., 2009). The desorption rate is assumed to be low in the Elovich model, and the desorption process may be irreversible. The kinetics of PQ adsorption on the β-CDSi composite best fit that model, thus confirming the data obtained. The parameter αin the Elovich equation indicates the initial adsorption coefficient. The γ-CDSi composite showed higher α, followed by β-CDSi and α-CDSi. On the other hand, the parameter β indicates the desorption rate of the process. In this regard, α-CDSi showed higher β than γ-CDSi and β-CDSi.

Based on the fit of the experimental data to the kinetic models, these results indicate the type of mechanism in the adsorption process. The adsorption of PQ on α-CDSi shows a good fit with the PFO and PSO models. The β-CDSi composite better fits the Elovich model, while the PQ adsorption process in γ-CDSi fits better with the PSO model. The above analysis suggests that PQ is adsorbed by physisorption and chemisorption mechanisms on the α-CDSi and γ-CDSi composites, in which physisorption is predominant. In contrast, the data obtained for PQ adsorption in the β-CDSi composite do not allow us to elucidate the type of mechanism. The ambiguity of fits to the PFO and PSO kinetic models has also been reported for removing methyltestosterone in β-CDSi (Bragança Carvalho et al., 2019). As with removing the steroid compound, the PQ adsorption process by CDSi composites in an aqueous medium may involve complexation with functionalized CDs through a physisorption mechanism. Thus, higher PQ association constants with the CD cavity induce an interpretation of the occurrence of a chemisorption mechanism. However, what governs the deposition of the adsorbate via the CD cavity are hydrophobic interactions, hydrogen interactions, and van der Waals forces (Bragança Carvalho et al., 2019). On the other hand, free carboxylic groups, remaining from the esterification agent, can confer an anionic character to the surface, favoring electrostatic interactions with the PQ molecule as pH increases and acting synergistically with the forces of interaction with the CD cavity (Wacławek et al., 2022).

**FIGURE 5:**
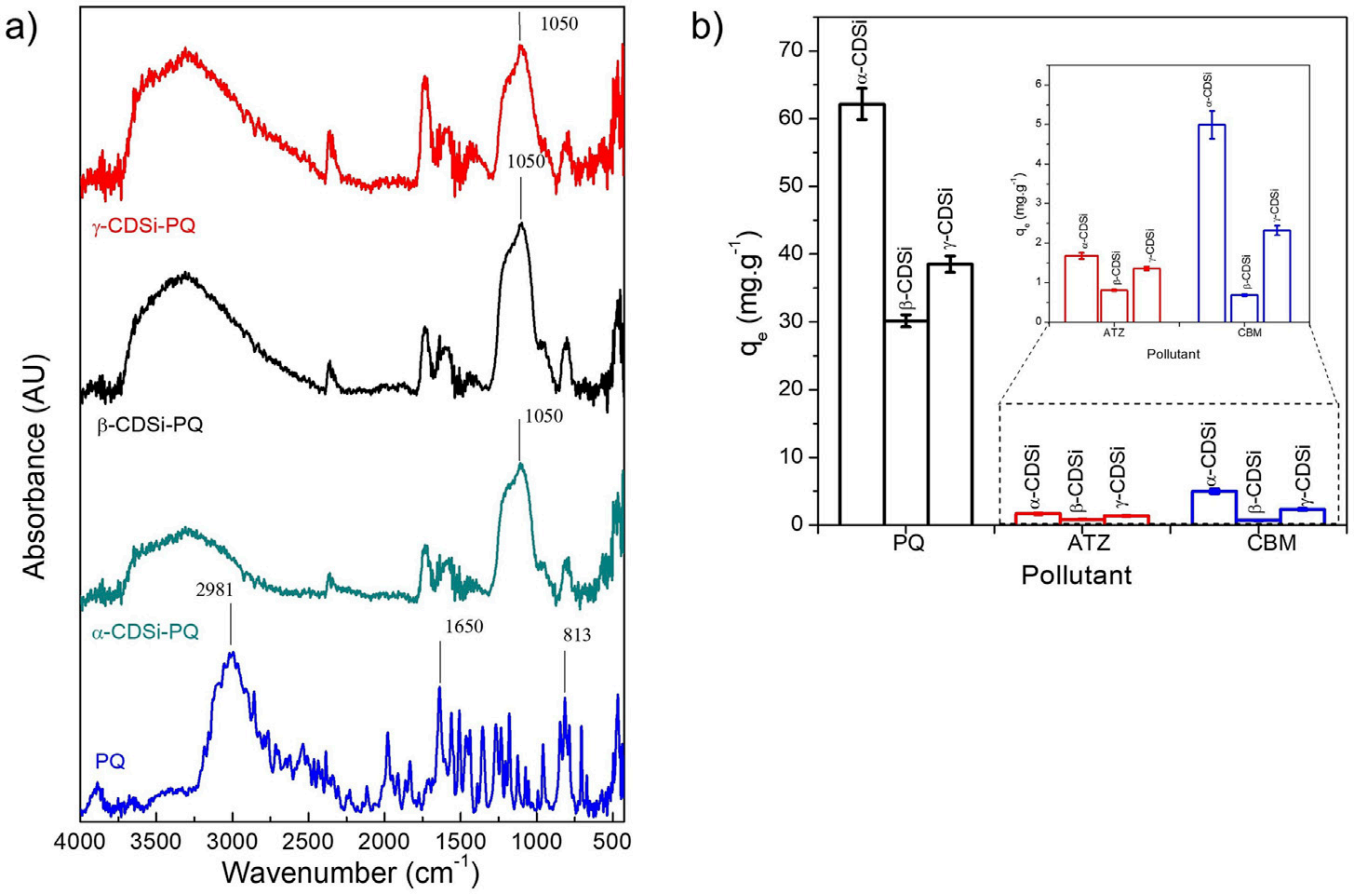
**(A)** FTIR spectra of PQ, α-CDSi-PQ, β-CDSi-PQ, and γ-CDSi-PQ. **(B)** Comparison of the equilibrium adsorption capacities of α-CDSi, β-CDSi, and γ-CDSi for the adsorption treatment of PQ (black), ATZ (red), and CBM (blue). Experimental conditions: [pollutant]_0_ 10 mg.L^−1^, T° 25°C, adsorbent dose, 0.02 %w/v, 8 ≤ pH ≤ 10.

After the PQ adsorption assays, the FTIR spectra of the CDSi-PQ complexes were analyzed. Figure 5A shows PQ, α-CDSi-PQ, β-CDSi-PQ, and γ-CDSi-PQ FTIR spectra. PQ shows a great diversity of signals (3,150–3,750 cm^−1^, 2,981 cm^−1^, 1,380–1,650 cm^−1^, 1,353 cm^−1^, 1,267 cm^−1^, 1,230 cm^−1^, 1,177 cm^−1^, and 813 cm^−1^) (Dinis-Oliveira et al., 2008; Baigorria and Fraceto, 2022c). C-H and C-C bond stretches of the aromatic ring of PQ are observed in the 1,267–813-cm^−1^ region. On the other hand, C-N bands can be observed at 1,650–1,570 cm^−1^. In the 3,100–3,750-cm^−1^ region, methyl bands belonging to the C-H strain of the PQ aromatic ring are observed (Dinis-Oliveira et al., 2008; Baigorria et al., 2023). The FTIR spectra of α-CDSi-PQ, β-CDSi-PQ, and γ-CDSi-PQ show a higher intensity and lower definition of the broadband from 3,700 to 2,750 cm^−1^, evidencing a change compared to the spectra before the removal of PQ in the CDSi composites. In addition, an increase in the intensity of the peaks at ∼1,050 cm^−1^ is observed in α-CDSi-PQ, β-CDSi-PQ, and γ-CDSi-PQ. There is also a multiplicity in several spectrum regions, indicating the presence of PQ in the CDSi composites after the removal treatment.

#### 3.2.2 Comparative analysis

CDSi composites synthesized in the present work, α-CDSi, β-CDSi, and γ-CDSi, exhibited equilibrium adsorption capacities of 87.36, 63.88, and 78.19 mg.g^−1^, respectively. Compared to the other adsorbent compounds of CDs reported in the literature, our results were better (Supplementary Table S2). Since the conditions compared for pH and temperature were similar, it is plausible to compare the adsorption results. We obtained adsorption capacities at equilibrium significantly higher than those reported, with substantially shorter adsorption equilibrium times. The high specific surface area value of the composites developed in the present work generates an important advantage, favoring the performance they develop as adsorbents of the studied pollutant.

Additionally, Supplementary Table S2 shows the results reported in the literature in recent years for other materials of different structures used as PQ adsorbents. Franco et al. (2022), in their review of the advances in removing the herbicide paraquat by adsorption technology, highlighted the lack of information found in the various works published in the literature, mainly in the experimental conditions used (Franco et al., 2022). There is also a scarce amount of work where PQ adsorption technologies are applied from water. Supplementary Table S2 shows an improvement in the results of *q*_*e*_ for NaY zeolites, carbon tubes, and Fe_3_O_4_@SiO_2_@SBA-3-SO_3_H (Rongchapo et al., 2017; Dehghani et al., 2021; Kouchakinejad et al., 2022). However, the initial doses of PQ used in these studies are irrelevant to applying the PQ removal treatments. As mentioned in Section 1, PQ concentrations between 0.03 and 30 mg.L^-1^ have been reported in watersheds (World Health Organization, 2018; World Health Organization, 2020). The works mentioned above, in which *q*_*e*_ values are higher than those reported in the present work, were conducted using initial PQ concentrations between 50 and 2,000 mg.L^−1^. This means that although the adsorption results are promising for these materials, the conditions studied are not feasible for their application in real environmental conditions. Moreover, considering the production costs of these adsorbent materials, they are also not conducive to their application. Based on the results found for CDs and other adsorbent materials compared to the CDSi materials developed in this work, α-CDSi, β-CDSi, and γ-CDSi are promising adsorbents for use in PQ removal treatments.

Preliminary studies to the present work allowed for analyzing the equilibrium adsorption capacities of CDSi against other agrochemicals of different chemical structures. Figure 5B shows the results of the adsorption of ATZ, CBM, and PQ using α-CDSi, β-CDSi, and γ-CDSi as adsorbents. The experimental conditions for the three pollutants studied (removal time, temperature, adsorbent dosage, initial pollutant concentration, and pH) were equal. The results show an evident difference in *q*_*e*_, being the highest for PQ removal. For the ATZ adsorption tests, *q*_*e*_ values of 1.68, 0.81, and 1.36 mg.g^−1^ were observed for α-CDSi, β-CDSi, and γ-CDSi, respectively, while for the CBM removal treatments, *q*_*e*_ values were 4.99, 0.69, and 2.321 mg.g^−1^ for α-CDSi, β-CDSi, and γ-CDSi, respectively. Structurally, the three contaminants studied, PQ, ATZ, and CBM, have different characteristics. In the working pH range, CBM is close to its pK_a2_ value and has an anionic charge in its structure (Baigorria and Fraceto, 2022c). In contrast, ATZ is a weak base/neutral molecule, so it does not exhibit charge variation at experimental pH (Ahmad and Rahman, 2009). As mentioned in previous sections, at 8 ≤ pH ≤ 10, the CDSi composites studied possess a negative charge on their surface. Due to this, considering that the highest number of interactions with contaminants occurs electrostatically, adsorption with PQ molecules is expected to be higher than for ATZ and CBM.

CDSi composites of similar structures to those developed in the present work were previously tested by the authors for removing methylene blue from contaminated waters. They found *q*_*e*_ of 47, 45, and 46 mg.g^−1^ for α-CDSi, β-CDSi, and γ-CDSi, respectively (2 ≤ pH ≤ 12, adsorbent dose 100 %w/v, [MB]_0_ = 50 mg.L^−1^) (Bragança Carvalho et al., 2014; Bragança Carvalho et al., 2022). As in the present work, the adsorption equilibrium time was longer for β-CDSi (4 h) than for α-CDSi and γ-CDSi (15 min). Observing these results, it is possible to affirm that the variation in the synthesis conditions of the CDSi composites allowed an improvement in the adsorption capacities of the materials. Then, comparing previous synthesis methodologies and performances of similar materials against other aqueous pollutants, we can say that the composites developed in this work have very promising results, thus indicating that the synthesized composites α-CDSi, β-CDSi, and γ-CDSi prove to be an excellent solution for the problem of aqueous contamination by pesticides and other aqueous pollutants.

## 4 Conclusion

Inorganic–organic hybrid materials were synthesized from CDs and Si with optimized synthetic processes. The CDSi composites were holistically characterized at the surface, compositional, and molecular levels. After functionalizing silica, CDSi hybrid composites with hydrophobic characteristics provided by CDs in the structure were obtained. Moreover, functionalization with CDs optimized the thermal properties, increasing the stability of the starting CDs and assigning high thermal stability to the CDSi composites. Inorganic–organic hybrid materials exhibited mesoporous surface characteristics. Environmental remediation tests for PQ showed better results for α-CDSi and γ-CDSi than for β-CDSi, with the results being dependent on the structural differences of the CDSi composites. Exothermic adsorptions with physisorption-governed mechanisms were observed, with small contributions from chemisorption for α-CDSi and γ-CDSi, compatible with interactions with the hydrophobic cavities of CDs. In addition, electrostatic-type interactions between the composites and PQ^2+^ were determined. The aqueous adsorption performance of α-CDSi, β-CDSi, and γ-CDSi composites *versus* ATZ and CBM was compared. The structural difference of the contaminants was observed to affect the adsorption efficiency of the CDSi composites, thus confirming the electrostatic-type interactions between the adsorbents and pollutants. Based on the results, optimizing the synthesis of the CDSi composites allowed the generation of hybrid materials with improved adsorption capacities, applicable to removing various aqueous pollutants such as agrochemicals like PQ. In addition, the performance against other pollutants was promising, indicating potential applications to other emerging contaminants. Thus, inorganic–organic hybrid materials like CDSi provide a possible and promising solution to societal problems, such as aqueous pollution.

## Data Availability

The original contributions presented in the study are included in the article/Supplementary Material; further inquiries can be directed to the corresponding author.
