# Supplementary material for: Cyclodextrin–silica hybrid materials: synthesis, characterization, and application in pesticide aqueous removal
Source: Front Chem. 2024 Aug 29;12:1450089. doi: 10.3389/fchem.2024.1450089 (PMC11390502; doi:10.3389/fchem.2024.1450089)
Supplement: Supplementary file 1 [file DataSheet1.PDF]

Supplementary Material

Cyclodextrin-silica hybrid materials: synthesis, characterization, and application in pesticide aqueous removal

Estefanía Baigorria[1,2*], Lucas Bragança Carvalho[1,3], Luciana Matos Alves Pinto[3], Leonardo Fernandes Fraceto[1]

[1]Institute of Science and Technology, São Paulo State University, Av. Três de Março 511, 18087–180 Sorocaba, SP, Brazil.

[2]Materiales Compuestos Termoplásticos (CoMP), Instituto de Investigaciones en Ciencia y Tecnología de Materiales (INTEMA), CONICET, Universidad Nacional de Mar del Plata (UNMdP), Av. Colón 10850, Mar del Plata, Buenos Aires, Argentina.

[3]Chemistry Department, Natural Sciences Institute, Universidade Federal de Lavras, Campus Universitário s/n, 37200–900 Lavras, MG, Brazil.

*** Correspondence:**
* Corresponding authors at: Baigorria Estefanía and Leonardo F. Fraceto.
E-mail addresses: esbaigorria@gmail.com (E. Baigorria), leonardo.fraceto@unesp.br (L.F. Fraceto).

Table S1. CP/MAS ^{13}C NMR (δ/ppm) for cyclodextrins (α and γ) free and functionalized.

	C-1	*C-4*	*C-2,3,5*	*C-6*	*Esther and/or carboxylic acid*	*CH_2 (ligand)*
α-CD	103.78	85.33	75.78	61.70		
	103.21	83.00	74.78	61.14		
	102.87	81.88	74.25			
	101.99	80.56	73.63			
	98.07	77.71	73.01			
			72.31			
			71.65			
mean	102.87	81.88	73.63	61.42		
α-CDSi	102.22	81.53	73.39	64.62	172.93	43.43
γ-CD	105.09	84.60	75.11	64.49		
	103.96	83.33	74.11	63.15		
	103.38	82.52	73.58	61.02		
	102.18	81.99	73.33	60.11		
	101.35	81.41	72.29			
	101.12	79.58	71.47			
	99.85	78.61	71.01			
	98.91	77.49				
	98.17	76.74				
mean	101.35	81.41	73.33	63.15		
γ-CDSi	102.63	82.31	73.97	64.91	173.1	44.20

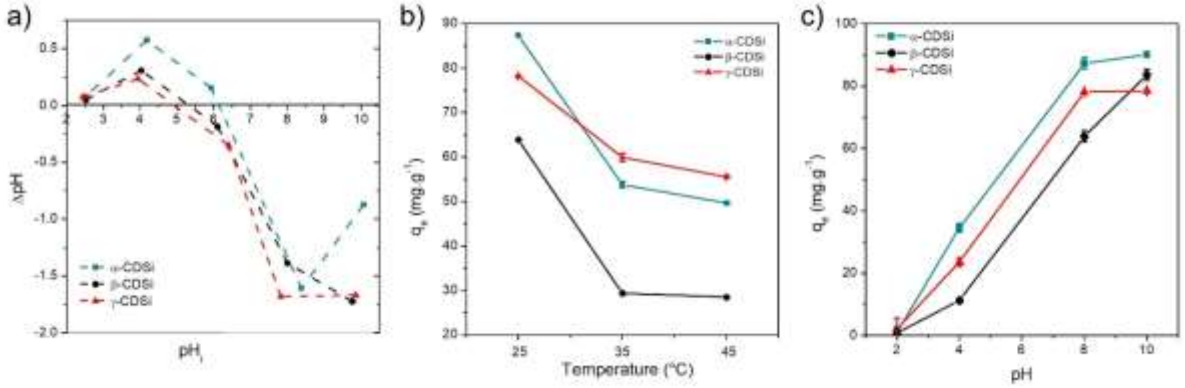

Figure S1. (a) PZC determination in CDSi composites samples: α-CDSi, β-CDSi, and γ-CDSi. (b) Temperature and (c) pH effect on the PQ adsorption ($[PQ]_0$= 20 mg.L^{-1}, adsorbent dose = 0.02 %w/v).

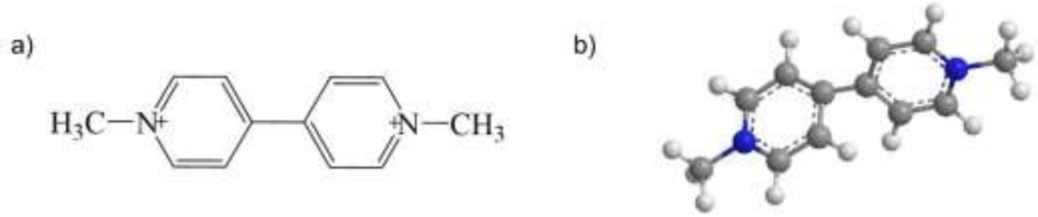

Figure S2. PQ structure (a) 2D, and (b) 3D.

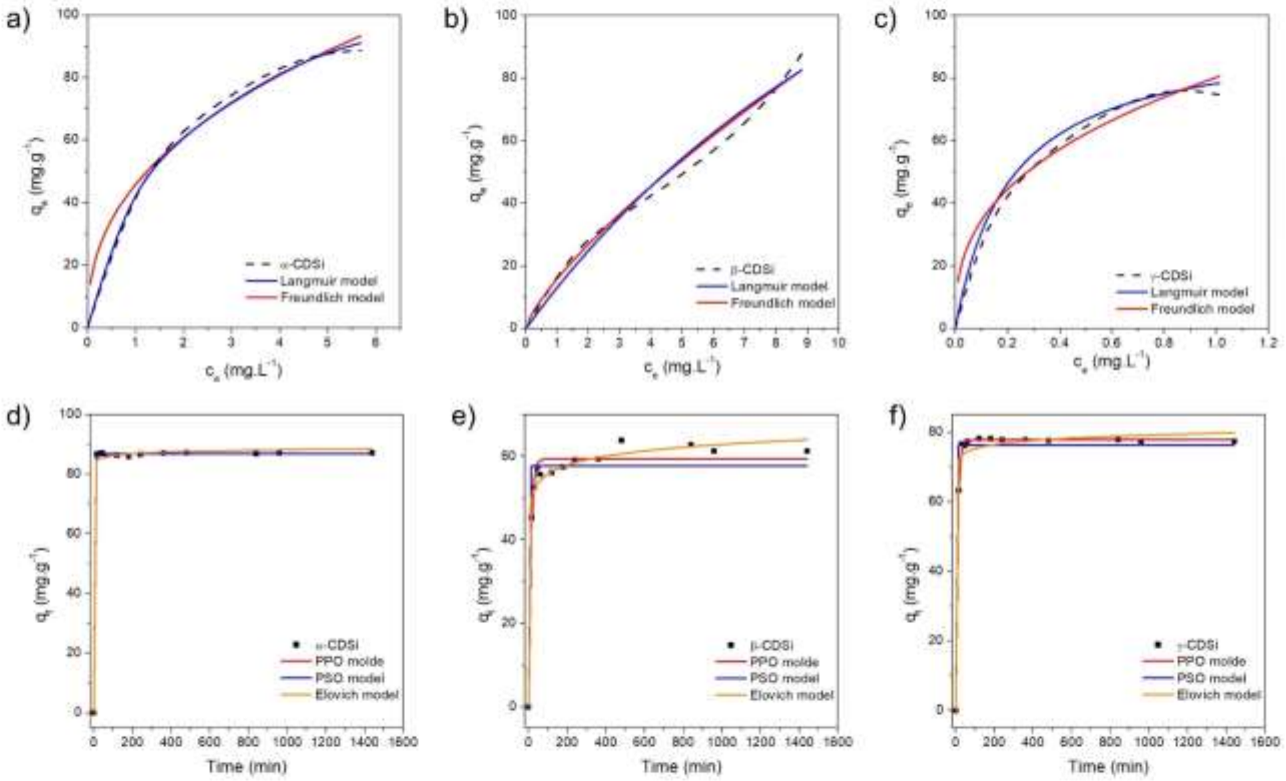

Figure S3. Non-linear fitting of PQ adsorption experimental data to the Langmuir and Freundlich isotherm models to the (a) α-CDSi, (b) β-CDSi, and (c) γ-CDSi composites. PFO, PSO and Elovich kinetic models to the (d) α-CDSi, (e) β-CDSi, and (f) γ-CDSi composites.

Table S4. Performance comparison of various PQ adsorption parameters reported for different CD and other materials reported: q_e, equilibrium removal time, temperature, mechanism, and dosage of PQ and adsorbent.

Materials	*q_e ($mg.g^{-1}$)*	*C_o ($mg.L^{-1}$)*	*Dose (%w/v)*	*T° (°C)*	*pH*	*S_{BET} ($m^2.g^{-1}$)*	*Equilibrium time (min)*	*$M^\#$*	*Reference*
CD MATERIALS									
β-cyclodextrin and 1,2,3,4-butanetetracarboxylic acid	10.8	25	0.2	30	8	0.15	120	L	(Junthip et al., 2019)
	19.7	50	0.2	30	8	0.15	120	L	(Junthip et al., 2019)
	25.8	200	0.2	30	8	0.15	120	L	(Junthip et al., 2019)
Cyclodextrin polymer crosslinked with citric acid	9.4	25	0.2	30	6.5	0.63	120	L	(Junthip, 2019)
	17.4	50	0.2	30	6.5	0.63	120	L	(Junthip, 2019)
	20.8	200	0.2	30	6.5	0.63	120	L	(Junthip, 2019)
	5	10	0.2	30	8	n.r.	360	L	(Junthip et al., 2018)

PET textiles coated with anionic cyclodextrin polymer	20.4	50	0.2	30	8	n.r.	360	L	(Junthip et al., 2018)
	25.9	250	0.2	30	8	n.r.	360	L	(Junthip et al., 2018)
Textile coated with anionic cyclodextrin polymer	4.5	10	0.2	30	6.5	n.r.	360	L	(Junthip et al., 2018)
	18.9	50	0.2	30	6.5	n.r.	360	L	(Junthip et al., 2018)
	23.7	250	0.2	30	6.5	n.r.	360	L	(Junthip et al., 2018)
Poly(vinyl alcohol)-cyclodextrin nanosponges	10.6	25	0.2	30	6.5	0.176	60	L	(Martwong et al., 2021)
b -Cyclodextrin nanosponges cross-linked with 1,2,3,4-butanetetracarboxylic acid and poly(vinyl alcohol)	12	25	0.2	30	8	n.r.	100	L	(Martwong et al., 2022a)
Cotton cord coated with 10 % b -cyclodextrin polymers	4.56	25	0.5	30	8	n.r.	200	L	(Martwong et al., 2022b)

Cotton cord coated with 5 % b-cyclodextrin polymers	4.75	25	0.5	30	8	n.r.	200	L	(Martwong et al., 2022b)
α-CDSi	**87.36**	**25**	**0.02**	**25**	**8-10**	**80**	**30**	**L-F**	**This work**
β-CDSi	**63.88**	**25**	**0.02**	**25**	**8-10**	**76**	**60**	**n.r.**	**This work** (Bragança Carvalho et al., 2019)
γ-CDSi	**78.19**	**25**	**0.02**	**25**	**8-10**	**26**	**30**	**L-F**	**This work**
OTHER MATERIALS									
Bentonite/zero valen iron	6.78	20	0.24	25	10	n.r.	1440	L	(Dehgani et al., 2020)
NaY Zeolite	234.4	1500	0.25	25	7	798	1440	L	(Rongchapo et al., 2017)
Carbon tubes	218.61	500	0.04	20	10	43.86	20	L	(Li et al., 2021)
Graphene oxide/mesoporous silice	29.15	24	0.08	25	7	134.33	1440	L	(Dehghani et al., 2021)
Bentonite/mesoporous silice	11.75	8	0.16	25	7	15.21	1440	L	(Rasaie et al., 2021)

Fe_3O_4@SiO_2@SBA-3-SO_3H	80	75	0.24	25	7	70.42	120	L	(Kouchakinejad et al., 2022)
FJS/InMOF	56.03	12	0.3	25	n.r.	n.r.	720	n.r.	(Sun et al., 2022)

[#]M: mechanism

*n.r.: non reported

References

Bragança Carvalho, L., Chagas, P. M. B., Marques, T. R., Razafitianamaharavo, A., Pelletier, M., Nolis, P., et al. (2019). Removal of the synthetic hormone methyltestosterone from aqueous solution using a β-cyclodextrin/silica composite. *J. Environ. Chem. Eng.* 7, 103492. doi: 10.1016/j.jece.2019.103492

Dehgani, Z., Sedghi asl, M., Ghaedi, M., Sabzehmeidani, M. M., and Adhami, E. (2020). Removal of paraquat from aqueous solutions by a bentonite modified zero-valent iron adsorbent. *New J. Chem.* 44, 13368–13376. doi: 10.1039/D0NJ02259D

Dehghani, Z., Sedghi-Asl, M., Ghaedi, M., Sabzehmeidani, M. M., and Adhami, E. (2021). Ultrasound-assisted adsorption of paraquat herbicide from aqueous solution by graphene oxide/ mesoporous silica. *J. Environ. Chem. Eng.* 9, 105043. doi: 10.1016/j.jece.2021.105043

Junthip, J. (2019). Water-insoluble cyclodextrin polymer crosslinked with citric acid for paraquat removal from water. *J. Macromol. Sci. Part A* 56, 555–563. doi: 10.1080/10601325.2019.1586444

Junthip, J., Jumrernsuk, N., Klongklaw, P., Promma, W., and Sonsupap, S. (2018). Removal of paraquat herbicide from water by textile coated with anionic cyclodextrin polymer. *SN Appl. Sci.* 1, 106. doi: 10.1007/s42452-018-0102-z

Junthip, J., Promma, W., Sonsupap, S., and Boonyanusith, C. (2019). Adsorption of paraquat from water by insoluble cyclodextrin polymer crosslinked with 1,2,3,4-butanetetracarboxylic acid. *Iran. Polym. J.* 28, 213–223. doi: 10.1007/s13726-019-00692-9

Kouchakinejad, R., Shariati, S., Abolhasani, J., Kalhor, E. G., and Vardini, M. T. (2022). Core-shells of magnetite nanoparticles decorated by SBA-3-SO3H mesoporous silica for magnetic solid phase adsorption of paraquat herbicide from aqueous solutions. *Colloids Surfaces A Physicochem. Eng. Asp.* 643, 128709. doi: 10.1016/j.colsurfa.2022.128709

Li, H., Qi, H., Yin, M., Chen, Y., Deng, Q., and Wang, S. (2021). Carbon tubes from biomass with prominent adsorption performance for paraquat. *Chemosphere* 262, 127797. doi: 10.1016/j.chemosphere.2020.127797

Martwong, E., Chuetor, S., and Junthip, J. (2021). Adsorption of Paraquat by Poly(Vinyl Alcohol)-Cyclodextrin Nanosponges. *Polymers (Basel).* 13. doi: 10.3390/polym13234110

Martwong, E., Chuetor, S., and Junthip, J. (2022a). Adsorption of Cationic Contaminants by Cyclodextrin Nanosponges Cross-Linked with 1,2,3,4-Butanetetracarboxylic Acid and Poly(vinyl alcohol). *Polymers (Basel).* 14. doi: 10.3390/polym14020342

Martwong, E., Sukhawipat, N., and Junthip, J. (2022b). Adsorption of Cationic Pollutants from Water by Cotton Rope Coated with Cyclodextrin Polymers. *Polymers (Basel).* 14. doi: 10.3390/polym14122312

Rasaie, A., Sabzehmeidani, M. M., Ghaedi, M., Ghane-Jahromi, M., and Sedaratian-Jahromi, A.

(2021). Removal of herbicide paraquat from aqueous solutions by bentonite modified with mesoporous silica. *Mater. Chem. Phys.* 262, 124296. doi: 10.1016/j.matchemphys.2021.124296

Rongchapo, W., Keawkumay, C., Osakoo, N., Deekamwong, K., Chanlek, N., Prayoonpokarach, S., et al. (2017). Comprehension of paraquat adsorption on faujasite zeolite X and Y in sodium form. *Adsorpt. Sci. Technol.* 36, 684–693. doi: 10.1177/0263617417715394

Sun, H.-Y., Gao, Y.-J., Li, J.-L., Zou, Y.-M., Feng, M.-L., and Huang, X.-Y. (2022). Removal of Toxic Dyes and Paraquat by a Dual-Functional Metal-Organic Framework. *ChemistrySelect* 7, e202203826. doi: 10.1002/slct.202203826
